# Frequency of Cryptosporidiosis in Children having Persistent Diarrhea

**DOI:** 10.12669/pjms.37.1.2700

**Published:** 2021

**Authors:** Mariam Danish Iqbal, Tahir Naeem, Umar Khurshid, Fatima Hameed

**Affiliations:** 1 Mariam Danish Iqbal, FCPS Microbiology. Pathology Department, Shalamar Medical & Dental College, Lahore, Pakistan; 2 Tahir Naeem, MCPS, D(ABMM). Pathology Department, Shalamar Medical & Dental College, Lahore, Pakistan; 3 Umar Khurshid, FCPS Microbiology. Microbiology Department, Armed Forces Institute of Pathology, Rawalpindi, Pakistan; 4 Fatima Hameed, FCPS Microbiology. Pathology Department, CMH Lahore Medical College, Lahore, Pakistan

**Keywords:** *Cryptosporidium*, Persistent diarrhea, Children

## Abstract

**Objective:**

Globally childhood diarrheal diseases continue to be the second leading cause of death. *Cryptosporidium spp* are important intestinal parasites that cause diarrhea in humans and animals particularly in developing countries. This investigation was carried out to find out the frequency of cryptosporidiosis in children presenting with persistent diarrhea.

**Methods:**

Two hundred stool samples were collected in this descriptive cross-sectional study conducted at Microbiology Department, Combined Military Hospital, Lahore Pakistan between the months of July to Dec 2014. Children aged five years to 12 years who presented with persistent diarrhea were included in the study. Stool specimens were processed using the modified acid-fast staining method, and microscopically examined for *Cryptosporidium* infection.

**Results:**

The average age of study participants was 7.95 with a standard deviation of 2.21 years. Among the participants 66% were males whereas 34% were females. Twenty eight percent had presence of oocysts in stool samples.

**Conclusions:**

The frequency of Cryptosporidiosis among children with persistent diarrhea was 28%. This high frequency indicates that this population is uniquely susceptible to infection. It also highlights the need for education about hygiene, accurate diagnosis, and treatment of Cryptosporidiosis. There is also a need for additional studies regarding the occurrence of this pathogen.

## INTRODUCTION

*Cryptosporidium* species are intracellular parasites that primarily infect the epithelial cells of the intestine, stomach, and sometimes biliary ducts.^1^ Genotypic studies have shown that although over twenty-two different species can cause disease in humans, only two species (*C. hominis* and *C. parvum*) cause most of the infections.^2^ It causes self-limiting gastroenteritis in normal as well as in immunocompromised individuals. This disease is characterized by abdominal cramps, vomiting, generalized malaise, weakness, fatigue, loss of appetite and nausea.^3^,^4^ It can lead to malnutrition, especially in children.^4^ According to World Health Organization, diarrhea is among the second most common cause of death in children.^5^ *Cryptosporidium* is among the more common causes of persistent diarrhea in children from developing countries and causes approximately one third of cases of pediatric diarrhea.^6^,^7^

The reservoirs of *Cryptosporidium* are both usually wild and domestic animals. It has been shown that in some parts of the world zoonotic *C. parvum* infection is due to in large part by infected calves. The usual mode of transmission of disease is through ingestion of contaminated drinking water and recreational water.^5^

It is ubiquitous and very resistant to chlorine at levels used in potable and swimming pool water, thus representing a major threat to community water supply. Nosocomial infections and daycare associated infections are common because of small ID50 dose, feco-oral transmission and prolonged environmental survival of this organism.^6^

It is proposed that infection of *Cryptosporidium* is established due to attachment of parasite to cells of host. The principal site of infection is always the intestinal tract. This protozoan results in impaired intestinal absorption followed by increased secretion into the gut lumen. Impairment in both humoral and cell-mediated immunity is involved. The entire life cycle of the parasite occurs in a single host and the oocysts are excreted in fully infective form.^7^

In children, Cryptosporidiosis is frequently related with diarrhea, malnutrition, cognitive problems and small growth. In half-starved children, the repeated infection of *Cryptosporidium* damages the mucosa of the gut resulting in further alteration in the absorption of nutrients and can also affect the growth rate of a child. Host behavioral factors also play an important role in the risk of cryptosporidiosis depending on the exposure to the pathogen.^8^

Therefore, a need to identify *Cryptosporidium* infection in a timely manner, which may prevent long hospital stay and exposure to other hospital acquired infections, is of paramount importance.^6^ If early diagnosis can be made, appropriate health plans can be implemented.

This type of study has not been conducted in Lahore before, specifically in the 5-12 years age group. A descriptive cross sectional study was therefore designed to find out the frequency of cryptosporidiosis in children presenting with persistent diarrhea in a tertiary care hospital.

## METHODS

The study was a descriptive cross-sectional study conducted at Microbiology Department, CMH, Lahore. Two hundred stool samples were collected from the months of July to December 2014, from children aged 5 years to 12 years who presented with persistent diarrhea. Sampling was non probability purposive. Questionnaire based on clinical and demographic data was filled by parents of participants. Children who had persistent diarrhea, that is, diarrhea lasting more than seven days with more than three episodes per day were included in the study.^5^ Children taking antibiotics or having any other enteric disease were excluded. Study was approved by Ethics Review Committee of Combined Military Hospital dated 30th October 2013, RTMC No. MIC-2011-058-122. Fecal material was concentrated by formalin-ethyl acetate sedimentation for getting maximum number of organisms. Slides were prepared and fixed with methyl alcohol, and oocysts of *Cryptosporidium* were identified in concentrated stool by modified Ziehl Neelson (ZN) staining (Kinyoun staining). *Cryptosporidium* was identified by its size (4-6 µm in diameter) and shape on modified ZN staining.^9^

*Cryptosporidium* oocysts appear bright red against a blue background in this method. Visualization of even one oocyst of *cryptosporidium* was considered as positive, and that patient considered to be infected.

### Data Analysis

Data were entered in computer software SPSS 20. Values of frequency of *Cryptosporidium* in stool specimens of children were calculated using formulae and expressed in percentages.


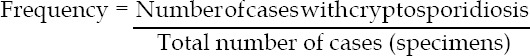


## RESULTS

The study included 200 participants. The average age of study participants was 7.95 with a standard deviation of 2.21 years. Among all the participants 66% (n= 132) were male whereas 34% (n=68) were female. Also among all the participants, 28% (n= 56) had presence of oocysts in stool sample. The relationship between gender and the presence of oocysts was insignificant according to the p value. (P=0.099). Eighty five percent of all the participants were those of admitted patients and 15% were of outdoor patients.

The control strain used from American type culture collection No 87714, *Cryptosporidium parvum*. The control strain was used to establish the validity of the oocysts that were identified, so as to make sure that artifacts were not confused with genuine oocysts.

## DISCUSSION

Since the 1980’s, *Cryptosporidium* has arisen as a pathogen to be taken notice of, especially in pediatric and immunocompromised patients. In developing countries, it is still not considered a serious threat, but it should be, because it may result in the development of cognitive and fitness problems that persist for years.^10^ This demands that the burden of the disease be documented and brought to the attention of the physicians especially pediatricians, so that they consider it among their list of differential diagnoses when dealing with children having persistent diarrhea.

According to our study the parasite identified in the stool was collected from mainly admitted children with mean age of 7.95 years, and increased distribution with more males than female. It has been shown by a study conducted in 2016 that *Cryptosporidium* is the main reason of diarrhea in children between the age range of six months to twelve years.^11^ It is proposed that high incidence of diarrhea in this age group may be due to lack of hygiene along with an immature immune system.^12^ Additionally, poor sanitation and contact with contaminated soil are also risk factors for the development of diarrhea.^13^

Because the protozoa was identified in the stool samples of a majority of hospitalized children, it is thought that nosocomial transmission of *Cryptosporidium* was also a cause of Cryptosporidiosis among children and staff of hospital, however this needs to explored further. Infection may be spread via polluted water or hands of people infected.^14^ Our study is in contrast to another study conducted in 2019 in Cameroon, that female children are more prone to develop cryptosporidiosis as compared to males. The reason of gender based difference is not clear as both male and female children have the same exposure to environment.^13^

We observed that the frequency of *Cryptosporidium* was found to be 28% in children aged from 5 - 12 years with persistent diarrhea. However a local study reported 9 % of the frequency of *Cryptosporidium* in children with age less than five years.^15^ A study conducted in Karachi in 2014 gave a 55% positivity rate of *cryptosporidium* in acute diarrhea.^16^ According to an Iranian study the frequency of *Cryptosporidium* in immunocompromised children was 4.16%.^17^ In another neighboring country, Iraq, the frequency is 22.27%, however this study includes both diarrheal and non diarrheal stool samples.^18^ Another study carried out in Mardan during the year of 2019 showed frequency to be 29.88% and the reason of this high prevalence may include unsafe water and poor sanitation.^4^ The present study had stool samples from chronic and persistent diarrhea, a factor not explored by other studies, as well as a selective age range, thus explaining the dissimilar frequency observed.

The increased frequency in our study (28%) indicates that *Cryptosporidium* is emerging as a real threat due to its pathogenic potential to cause severe morbidity and, thus should be considered in all cases with prolonged diarrhea. This is true for developing countries like Pakistan, where water supplies are almost always compromised and the safety of drinking water is constantly under suspicion, due to the fact that this parasite is very hard to destroy in water.^19^,^20^ There is also the theory that perhaps increased frequency could be due to the fact that the children were already in a immunocompromised state due to the prolonged diarrhea, and as an article published in India states that, as we age *Cryptosporidium* infection seems to become more common. It also indicates that certain regions are more endemic than others for *cryptosporidium*, especially in developing countries.^21^

### Limitation of the study

Identification of *Cryptosporidium* is only carried out by microscopy which can overestimate or underestimate the incidence. Due to the lack of advanced techniques of PCR and ELISA, the comparison of frequency or prevalence was carried out only with studies who used microscopy for detection.

## CONCLUSIONS

The stool samples collected were from chronic and persistent states which is the most likely reason of the high frequency of oocysts. There should be routine testing of stool for *Cryptosporidium* when acute diarrhea presents so as to prevent the chronicity. Additional studies are needed to find out the issues related with the occurrence of this parasite. There is also a need of laboratory-based studies for estimation of *Cryptosporidium* related diseases.

### Authors’ Contribution:

**MDI:** Conceived, designed, did statistical analysis,editing of manuscript and is responsible for integrity of research.

**UK and FH:** Did data collection and manuscript writing.

**TN:** Did review and final approval of manuscript.

## References

[1] Leitch GJ, He Q (2012). Cryptosporidiosis - an overview. J Biomed Res.

[2] Miyamoto Y, Eckmann L (2015). Drug Development against the Major Diarrhea-Causing Parasites of the Small Intestine Cryptosporidium and Giardia. Front Microbiol.

[3] Quadri F, Nasrin D, Khan A, Bokhari T, Tikmani SS, Nisar MI (2013). Health care use patterns for diarrhea in children in low-income periurban communities of Karachi, Pakistan. Am J Trop Med Hyg.

[4] Khan A, Shams S, Khan S, Khan MI, Khan S, Ali An (2019). Evaluation of prevalence and risk factors associated with *Cryptosporidium* infection in rural population of district Buner Pakistan. PLoS One.

[5] World Health Organization. Water Sanitation Health (2011). Guidelines:Drinking Water Quality.

[6] Mohammad A, Dagefu H, Jilo K (2017). Cryptosporidium and Its Public Health Importance:Review. Int J Res Studies Micro Biotechnol.

[7] Butt T, Ahmad RN, Kazmi SY, Afzal RK, Leghari MJ (2018). Cryptosporidiosis in a case of celiac disease. Pak Armed Forces Med J.

[8] Bouzid M, Hunter PR, Chalmers RM, Tyler KM (2013). Cryptosporidium pathogenicity and virulence. Clin Microbiol Rev.

[9] Center for Disease Control and Prevention (2013). Lab identification of Parasites of Public Health Concern Cryptosporidiosis.

[10] Al-Shehri H, Stanton MC, LaCourse JE, Atuhaire A, Arinaitwe M, Wamboko A (2016). An extensive burden of giardiasis associated with intestinal schistosomiasis and anaemia in school children on the shoreline of Lake Albert, Uganda. Trans R Soc Trop Med Hyg.

[11] Brunet J, Lemoine JP, Pesson B, Valot S, Sautour M, Dalle F (2016). Ruling out nosocomial transmission of Cryptosporidium in a renal transplantation unit:Case report. BMC Infect Dis.

[12] Syed S, Ali A, Duggan C (2016). Environmental Enteric Dysfunction in Children. J Pediatr Gastroenterol Nutr.

[13] Tombang AN, Ambe NF, Bobga TP, Nkfusai CN, Collins NM, Ngwa SB (2019). Prevalence and risk factors associated with cryptosporidiosis among children within the ages 0-5 years attending the Limbe regional hospital, southwest region, Cameroon. BMC Public Health.

[14] Ghoshal U, Dey A, Ranjan P, Khanduja S, Agarwal V, Ghoshal UC (2016). Identification of opportunistic enteric parasites among immunocompetent patients with diarrhoea from Northern India and genetic characterisation of *Cryptosporidium* and Microsporidia. Indian J Med Microbiol.

[15] Mumtaz S, Ahmed J, Ali L (2010). Frequency of *Cryptosporidium* infection in children under five years of age having diarrhea in the North West of Pakistan. Afr J Biotechnol.

[16] Ali S, Mumar S, Kalam K, Raja K, Baqi S (2014). Prevalence clinical presentation and treatment outcome of cryptosporidiosis in immunocompetent adult patients presenting with acute diarrhoea. J Pak Med Assoc.

[17] Hazrati TK, Barazesh A, Hajazi S, Mostaghim M (2011). Prevalence of *Cryptosporidium* in children referred to oncology center of Imam Khomeini hospital in Urmia Iran. Pak J Med Sci.

[18] Al-Saeed AT, Abdo JM, Gorgess RG (2020). Cryptosporidiosis in Children in Duhok City / Kurdistan Region / Iraq. J Pak Med Assoc.

[19] Rossle NF, Latif B (2013). Cryptosporidiosis as threatening health problem:A review. Asian Pac J Trop Biomed.

[20] Gerace E, Lo Presti VDM, Biondo C (2019). *Cryptosporidium* Infection:Epidemiology Pathogenesis, and Differential Diagnosis. Eur J Microbiol Immunol (Bp).

[21] Manocha H, Dua S, Chander Y, Tailang M (2014). Cryptosporidiosis whether it is more prevalent in Southern India. Trop Parasitol.

